# A fatal case report of sepsis caused by *Robinsoniella s*p.

**DOI:** 10.1590/S1678-9946202668022

**Published:** 2026-03-02

**Authors:** Meijia Huang, Hongjuan Zhang, Xinyue Li, Xiangchentao Zhang, Yunmin Xu

**Affiliations:** 1 Kunming Medical University, The First Affiliated Hospital, Department of Clinical Laboratory, Kunming, Yunnan, China; 2 Yunnan Key Laboratory of Laboratory Medicine, Kunming, Yunnan, China; 3 Chuxiong Prefecture Center for Disease Control and Prevention, Chuxiong, Yunnan, China; 4 Mengzi City Hospital of Traditional Chinese Medicine, Mengzi, Yunnan, China

**Keywords:** *Robinsoniella* species, Sepsis, Anaerobic bacteria, 16S rRNA

## Abstract

*Robinsoniella* species are anaerobic, spore-forming, Gram-positive bacilli that are rarely associated with human infections because of their slow growth and the limitations of conventional identification methods. We describe a fatal case of sepsis in an 84-year-old woman with hypertension, diabetes, and chronic renal insufficiency. During hospitalization, she developed impaired consciousness, polymicrobial infections, and multiple organ failure. Despite aggressive antimicrobial and supportive treatment, her condition deteriorated, leading to death. This study identified a *Robinsoniella* isolate (designated Rp5645) by 16S rRNA gene sequencing, showing 98.35% similarity to *R. peoriensis* PPC31. As this value falls below the accepted species-level threshold, Rp5645 may represent a novel *Robinsoniella* species. Unfortunately, the isolate lost viability before further genomic or phenotypic studies could be performed. This outcome illustrates the technical challenges in recovering and preserving fastidious anaerobes and underscores the crucial role of rapid molecular identification in confirming rare pathogens. This case broadens the clinical spectrum of *Robinsoniella* infections and highlights its potential pathogenic capacity, particularly in older or immunocompromised patients. It also emphasizes the need for timely molecular characterization to prevent the loss of valuable data from uncommon clinical isolates.

## INTRODUCTION

*Robinsoniella* species belongs to the phylum *Firmicutes*, class *Clostridia*, order *Clostridiales*, and family *Lachnospiraceae*. It is a strictly anaerobic gram-positive spore-forming bacillus ^1^. Cotta *et al*.^2^ first isolated and described the type species *Robinsoniella peoriensis* in 2009 from a swine manure storage pit and a human blood specimen, classifying it within the *Clostridium* cluster XIVa. Although *R. peoriensis* has been detected in environmental and animal intestinal samples, including soil^3^, wood turtle^4^, and *Neophocaena asiaeorientalis asiaeorientalis* ^5^, infections in humans remain extremely rare. Sporadic case reports have indicated that it can cause diverse infections such as osteoarticular infections^6,7^, bacteremia^8–16^, infective endocarditis^17^, and peritonitis^11^. Because of its slow growth and limited representation in commonly used identification systems (e.g., VITEK and MALDI-TOF), clinical laboratories often misidentify or overlook this organism^18^. Most reported cases involve patients with an underlying disease or immune compromise, characterized by rapid disease progression and poor prognosis. To date, the clinical features, pathogenic mechanisms, and optimal therapeutic strategies for *R. peoriensis* infections remain unclear. This study describes a fatal case of sepsis caused by a *Robinsoniella* species isolate closely related to *R. peoriensis*. This case contributes to our understanding of the potential pathogenic role of *Robinsoniella* species and highlights the diagnostic importance of molecular identification in rare anaerobic infections.

### Ethics

This study was approved by the Medical Ethics Committee of the First Affiliated Hospital of Kunming Medical University (approval N° 257). Informed consent was obtained from the patient.

## CASE REPORT

An 84-year-old woman presented with altered consciousness lasting for one day. According to her family, she developed progressive unresponsiveness without an apparent trigger—characterized by drowsiness, sluggish reactions, and productive cough with yellow purulent sputum. She showed no chest pain, dyspnea, or fever with chills. After symptom onset, the patient was brought to the emergency department of our hospital via 120 emergency medical services for evaluation and treatment. On admission, non-contrast chest computed tomography revealed patchy low-density shadows within the bronchi of both lower lobes and consolidation and partial atelectasis in the lower lungs. The upper and right middle lobes showed mild inflammatory exudates had occurred. Her medical history included >20 years of hypertension, a long-standing horseshoe kidney, and previous surgeries for renal calculi and bilateral cataract extraction. Specific medications and surgical details were unavailable. Upon physical examination, the patient appeared frail and drowsy, with a Glasgow Coma Scale score of E4VTM6. Her vital signs, including blood pressure, pulse rate, and respiratory rate, were temporarily stable.

After admission, laboratory investigations found a white blood cell count of 14.61 × 10^9^/L with 94.40% neutrophils. High-sensitivity C-reactive protein totaled 29.80 mg/L; procalcitonin, 0.21 ng/L; and interleukin-6, 12.24 pg/mL. Serum albumin decreased to 28.2 g/L. Electrolyte analysis showed 3.48 mmol/L of potassium, 150.30 mmol/L of sodium, 115.10 mmol/L of chloride, 2.10 mmol/L of calcium, 0.73 mmol/L of magnesium, and 0.61 mmol/L of inorganic phosphate. Coagulation studies showed 5.2 mg/L of fibrinogen degradation products, 1.51 mg/L of D-dimer, 4.20 g/L of fibrinogen, 13.6 s prothrombin time, and 33.3 s activated partial thromboplastin time. Immunological testing found an absolute lymphocyte count of 449/μL, with T lymphocytes accounting for 48.35%, Th cells for 17.41%, and NK cells for 42.06%.

Cultures of lower respiratory tract specimens yielded carbapenem-resistant *Pseudomonas aeruginosa*, with imipenem and meropenem MIC values ≥16 μg/mL. *Enterococcus faecium* was isolated from the urine samples. A combined antimicrobial therapy with amikacin and vancomycin was initiated according to these findings. Subsequently, an anaerobic blood culture was positive. Despite intensive antimicrobial treatment and comprehensive supportive care, the patient's condition progressively deteriorated, leading to multiple organ failure and severe complications including gastrointestinal bleeding. Ultimately, the patient succumbed to the illness despite resuscitation.

**Figure 1:**
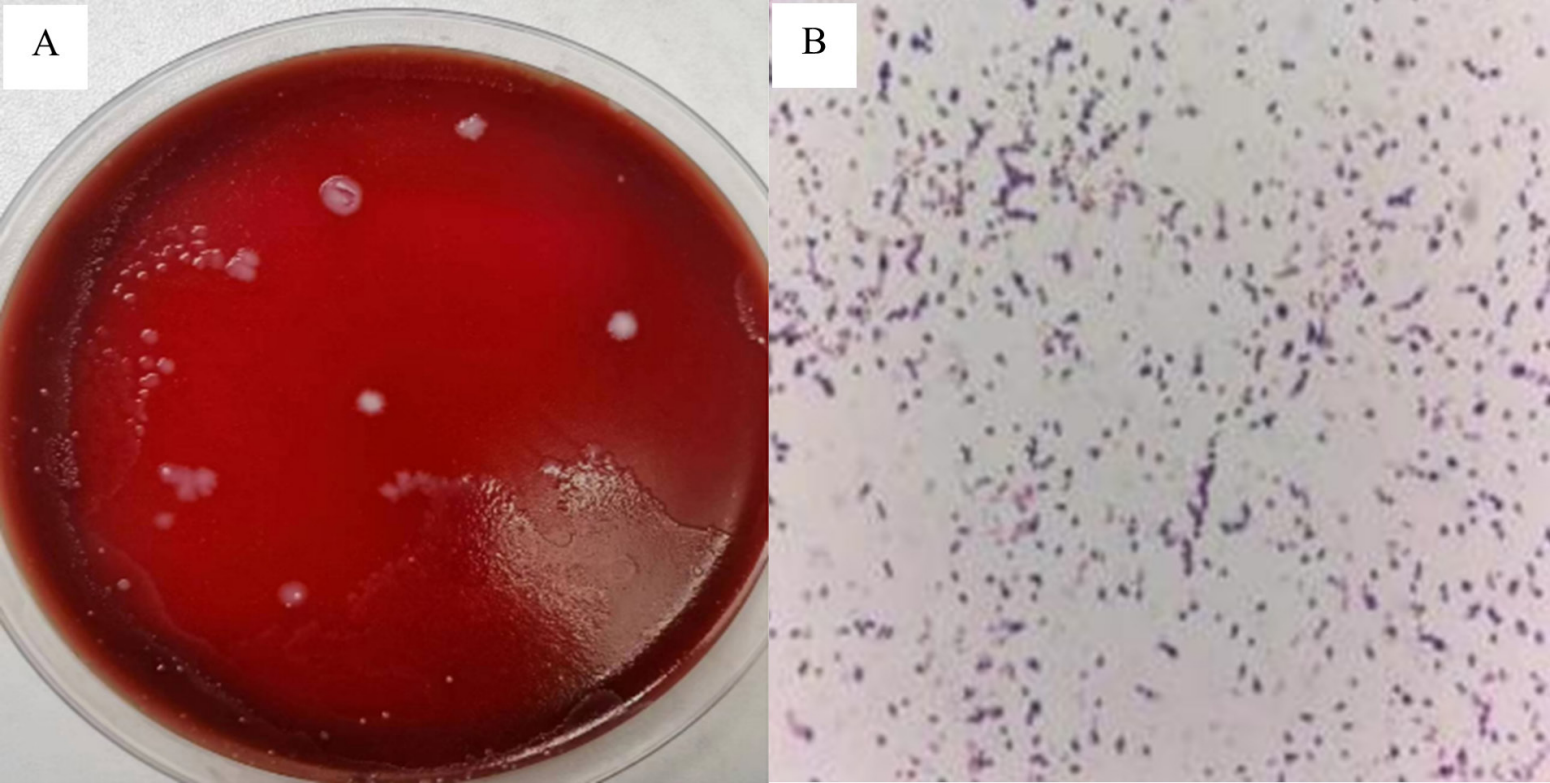
Colony and microscopic morphology of *Robinsoniella peoriensis*: (A) Flat white colonies with mixed sizes and smooth edges grown on anaerobic blood agar; (B) Gram-positive rod-shaped bacteria observed under a microscope (×1000).

Positive blood culture specimens were subcultured on Columbia blood agar plates and incubated anaerobically for 48 h. This yielded grayish-white non-hemolytic colonies of varying sizes with smooth edges. Gram staining found gram-positive, oval-shaped short rods with a few spores under microscopy (Figure 1). Identification using the MALDI-TOF mass spectrometry system failed to yield a reliable result. Therefore, the isolate was further submitted to Shanghai Sangon Biotech Co., Ltd. for partial 16S rRNA gene sequencing. EZBioCloud 16S-based identification indicated that the isolate from this case, designated strain Rp5645 (GenBank accession N° PX462034), showed the highest similarity to *Robinsoniella peoriensis* PPC31 (GenBank accession N° NR_041882.1), with a 16S rRNA gene sequence similarity of 98.35%. This value is below the 98.65% similarity threshold for species distinction suggested by Kim *et al*.^19^, indicating that strain Rp5645 may represent a putative novel species within the genus *Robinsoniella*. Therefore, we refer to the organism as "*Robinsoniella* sp." pending further taxonomic validation. Unfortunately, the isolate lost viability during preservation, precluding additional genomic, phenotypic, or antimicrobial susceptibility studies.

## DISCUSSION

*Robinsoniella* species are rarely associated with human infections. Most documented cases have involved *R. peoriensis*. Clinical documents include bloodstream^8–16^, endocarditis^17^, osteoarticular^6,7^, and intra-abdominal manifestations^11^. Most reported patients present with polymicrobial infections and underlying immunocompromised conditions.

The patient in this report was an older female adult with multiple predisposing conditions, including long-standing hypertension, horseshoe kidney, and chronic renal insufficiency. She was immunocompromised and developed polymicrobial infection. This comorbidity weakens the host's defense integrity, enabling opportunistic pathogens to gain access to the bloodstream. As the disease progressed, the patient experienced multiple organ failure and gastrointestinal bleeding, which led to death. Consistent with previously reported cases (Table 1), this case showed a rapidly progressive clinical course and poor prognosis.

**Table 1:** Cases associated with *Robinsoniella peoriensis* bacteremia.

Article	Year	Country	Gender	Age	Disease	Treatment	Outcome
López *et al*.^9^	2010	Spain	Male	50	Alcoholic cirrhosis	MTZ, CIP	Death
Shen *et al*.^10^	2010	China	Male	42	Pancreatic carcinoma	MTZ	Death
Gomez *et al*.^11^	2011	America	Female	79	Myocardial infarction	TZP, LEV, MTZ	Death
Jeon *et al*.^12^	2012	South Korea	Male	76	Aspiration pneumonia	VA, MEM, SXT	Death
Lim *et al*.^13^	2017	South Korea	Male	63	Small cell lung cancer	TZP, IPM	Death
Ursenbach *et al*.^14^	2020	France	Female	79	Endocarditis	AMX, DOX	Healthy
Yang *et al*.^8^	2021	China	Male	73	Prostate tumor	IPM	Healthy
Mejia-Gomez *et al*.^15^	2022	Canada	Female	47	Endometrial carcinoma	TZP, DOX	Healthy
Furuya *et al*.^16^	2023	Japan	Female	84	Peritoneal carcinoma	TZP, VA, MEM	Healthy
This study	2023	China	Female	84	Septicemia and severe pneumonia	AMK, IPM, VA	Death

MTZ = metronidazole; CIP = ciprofloxacin; TZP = piperacillin-tazobactam; VA = vancomycin; MEM = meropenem; SXT = co-trimoxazole; IPM = imipenem; AMX = Amoxicillin; DOX = doxycycline; AMK = Adderall Amikacin.

Identifying *Robinsoniella* species remains a diagnostic challenge. This organism grows slowly, requires strictly anaerobic conditions, and produces colonies lacking distinctive morphological characteristics. Most conventional automated identification systems, such as VITEK 2 and MALDI-TOF MS, often result in "unidentified" outcomes or misidentification as a member of the Clostridium genus.

In recent years, advances in sequencing technologies have played pivotal roles in the identification and characterization of uncommon or fastidious pathogens. Based on 16S rRNA gene analysis, strain Rp5645 shared 98.55% sequence similarity with *R. peoriensis* PPC31, slightly below the accepted cutoff for species delineation. This finding suggests that the isolate may represent a previously unrecognized member of the genus *Robinsoniella*. Although the strain unfortunately lost viability and was unable to undergo further phenotypic or genomic characterization, this case still provides valuable insights into the potential pathogenic spectrum of *Robinsoniella* species. To our knowledge, reports of human infections caused by this genus remain exceedingly rare, making this observation a meaningful addition to the limited literature. Moreover, the loss of the viable strain underscores the practical challenges in preserving fastidious anaerobes and highlights the necessity of prompt molecular identification and cryopreservation of rare clinical isolates. Strengthening such practices will facilitate future genomic, taxonomic, and therapeutic investigations, improving our understanding of these uncommon pathogens and their clinical significance.

Regarding antimicrobial susceptibility and treatment, the available data remain limited, and no standardized therapeutic regimen has been established for *Robinsoniella* bloodstream infections. Moreover, official antimicrobial susceptibility breakpoints for this species are yet to be defined. Reference can be made to the antimicrobial susceptibility breakpoints guidelines of the Clinical and Laboratory Standards Institute^20^ for guidance. Previous reports have suggested that *R. peoriensis* infections may respond to agents such as piperacillin–tazobactam, metronidazole, or carbapenems^15^. In this case, the patient's critical condition prohibited antimicrobial susceptibility testing. Despite empirical combination therapy with amikacin and vancomycin, infection control is unsatisfactory, emphasizing that early pathogen identification and targeted antimicrobial treatment are crucial determinants of clinical outcomes.

Overall, *Robinsoniella* infections are rarely recognized in clinical practice, are difficult to diagnose, and lack standardized therapeutic experience. Although *Robinsoniella* is an uncommon pathogen, it can cause severe or even fatal infections in high-risk patients. Therefore, strengthened collaboration between clinical and laboratory teams is essential along with the application of molecular identification or gene sequencing techniques for anaerobic isolates that are difficult to apply using conventional methods. Accumulating additional case data and antimicrobial susceptibility information will help inform future diagnostic and therapeutic strategies against this emerging pathogen.

## CONCLUSION

*Robinsoniella*, although rarely encountered, can cause severe infections, particularly in immunocompromised individuals. This fatal case of septicemia highlights their possible pathogenic role in high-risk patients and the diagnostic limitations of conventional methods for uncommon anaerobes. Sequencing-based identification provided crucial taxonomic insight, evincing a strain likely representing a novel *Robinsoniella* species. The experience emphasizes the importance of preserving rare clinical isolates to enable future genomic and phenotypic studies, which are essential for accurate classification and improved understanding of their clinical significance.

## Data Availability

The complete anonymized dataset supporting the findings of this study is included within the article itself.
